# Successful student learning outcomes in Moroccan higher education: Causal configurations of pedagogical, motivational, and ICT conditions using fuzzy-set Qualitative Comparative Analysis (fsQCA)

**DOI:** 10.1371/journal.pone.0347120

**Published:** 2026-04-21

**Authors:** Razzouki mustapha, Wided Ragmoun, Salah Ben Hammou

**Affiliations:** 1 Faculty of Economics and Management, Sultan Moulay Slimane University, Beni Mellal, Morocco; 2 Department of Business Administration, College of Business and Economics, Qassim University, Buraydah, Saudi Arabia; 3 Faculty of Languages, Arts and Human Sciences, Hassan 1st University, Settat, Morocco; BRAC Business School, BRAC University, BANGLADESH

## Abstract

As the demand for better educational quality and improved student performance grows, institutions face increasing challenges in making their teaching methods more effective to ensure successful learning outcomes. Most practical recommendations tend to focus on isolated effects, but learning success usually results from interconnected factors that work together. Drawing on constructivist Learning Theory, this study examines pedagogical and motivational factors that can positively impact learning results in higher education within Moroccan universities. The main goal is to identify and evaluate the nonlinear individual effects and the interactive causal configurations involving multiple conditions, such as teacher motivation, pedagogical leadership, self-efficacy, instructional innovation, ICT use, and student motivation, on learning outcomes. Empirical data were gathered through a questionnaire completed by 349 Moroccan university students, using measurement scales for key variables. The analysis employed the fuzzy-set Qualitative Comparative Analysis (fsQCA) method, which helps identify different combinations and causal pathways that lead to high academic achievement. Findings suggest that excellent learning outcomes are not caused by a single factor but by the interaction of several interdependent conditions. Different “recipes” can produce similar strong results. Certain combinations, especially those with strong teacher motivation, effective leadership, and strategic ICT use, proved to be reliable configurations, showing that technology is most impactful when integrated into supportive pedagogical and motivational environments. These results can help redefine and evaluate integrated educational policies, considering the complex interactions among individual, pedagogical, and technological factors. This approach promotes more effective and equitable learning environments through coherent bundles of mutually reinforcing interventions, instead of isolated efforts. Ultimately, this research enables educators and policymakers to develop more targeted strategies, utilize resources more efficiently, and improve overall educational effectiveness.

## 1. Introduction

In higher education, learning outcomes specify what students should learn and demonstrate upon successfully completing a course or programme [1,2]. They represent the intended outcomes of the learning process, particularly in terms of skills and knowledge acquisition, and are used to evaluate academic performance [3]. In practice, these outcomes have become a shared reference point for students, instructors, and institutions, informing curriculum design, classroom implementation, and quality assurance processes in increasingly accountable higher education environments. To achieve these learning outcomes, students must be provided with specific experiences and supportive conditions. In recent years, there has been growing interest in academic performance amid profound transformations in educational systems, and improving student learning outcomes has become a central concern for both public policymakers and education practitioners [4]. These transformations include the intensification of digital learning, rising expectations regarding employability-related competencies, and a stronger emphasis on evidence-based teaching, which together make the question of how to secure strong learning outcomes both timely and practically consequential. Academic achievement is no longer solely dependent on a learner’s intelligence or motivation; instead, it results from a complex interplay of pedagogical, institutional, and technological factors [5].

Existing research has predominantly focused on these factors, such as teacher-related attributes and behaviours. Teacher motivation, pedagogical leadership, perceived self-efficacy, and the ability to implement innovative teaching practices are recognised as key drivers of student achievement [6,7]. These individual factors are often amplified by collective dynamics and environments that foster innovation [8]. Simultaneously, students’ expectations and intrinsic motivation play crucial roles, especially in learning contexts that promote autonomy and active engagement [9,10,11]. Taken together, prior studies strongly suggest that both teacher-related resources (for example, efficacy and innovation capacity) and student-related resources (for example, intrinsic motivation) matter, yet they also imply that these conditions may operate differently depending on how they co-occur within a given learning setting [12].

However, despite extensive studies trying to identify and define determinants and factors, the majority of previous studies dealt with these factors considered them as independent with a linear effect, limited attention has been paid to identify the configurations of conditions that significantly affect academic performance or the relative importance of an exhaustive approach in this context to maximise students’ outcomes, success or performance. This reveals a significant gap in our understanding of such a nonlinear and dynamic approach. In fact, this topic has become a pressing priority for educational researchers [13]. More specifically, the current gap is not the absence of evidence on “what matters”, but the limited clarity on “what works together”: the literature still rarely explains how multiple pedagogical and motivational conditions combine to produce high learning outcomes, or whether different combinations can lead to the same level of success across heterogeneous higher education contexts [14,15]. This integrated perspective challenges traditional linear models that isolate variables, advocating a combinatorial analysis better aligned with the complexities of real-world educational environments [16]. A key research question emerges: How do these elements interact to produce successful learning outcomes?

In terms of methodological approach, recent studies have increasingly focused on multi-conditional approaches, such as Qualitative Comparative Analysis (QCA), which extend beyond simple correlations by identifying relevant causal configurations [17,18]. This method, particularly suited for studying complex phenomena, enables the detection of multiple equifinal pathways leading to the same outcome [14]. In the context of higher education, applying fuzzy-set Qualitative Comparative Analysis (fsQCA) offers valuable insights into how specific combinations of teacher and student-related factors are linked to high learning performance [15]. This methodological framework deepens our understanding of systemic interactions within educational processes, transitioning from isolated factor analysis to more holistic interpretations. Accordingly, the novelty of the present study lies in explicitly shifting the unit of explanation from net effects to configurations, which allows us to capture equifinality and causal asymmetry that are difficult to observe with conventional linear models [17].

Based on this, the present study aims to investigate the combined effects of teacher-related factors (motivation, leadership, efficacy, pedagogical innovation, and ICT use) and student motivation on high academic performance. Utilising data collected from a sample of university students and analysed through fsQCA, this research seeks to identify the specific combinations of factors that contribute to student learning outcomes. The anticipated results are expected to inform training policies, promote the adoption of innovative teaching approaches, and provide concrete recommendations to enhance teacher development and improve student engagement. This research contributes to the ongoing conversation about educational effectiveness by presenting a configurational viewpoint on the elements that foster successful learning outcomes in higher education. Utilising the fuzzy-set Qualitative Comparative Analysis (fsQCA) method, this study extends beyond conventional linear models to reveal various equally effective combinations of factors, including instructional strategies, institutional support, student engagement, and assessment practices, that contribute to high academic performance. The results provide both theoretical and practical contributions. Theoretically, it deepens our understanding of the complex causal relationships within educational settings. In practice, it offers actionable configurations that policymakers, administrators, and educators can implement to improve student success across diverse institutional environments. Following this introduction, the article is structured as follows: Section 2 reviews the relevant literature; Section 3 outlines the research methodology; and Section 4 presents and discusses the main findings, concluding with implications, limitations, and recommendations for future research.

## 2. Theoretical framework

### 2.1 Constructivist Learning Theory

Constructivist Learning Theory (CLT) asserts that learners actively construct knowledge through their engagement with the environment, past experiences, and social interactions, rather than passively absorbing information [19,20]. Its key principles directly relate to active learning, contextualization, social interaction, scaffolding, and learner reflection, emphasising that understanding develops from meaningful experiences and collaborative activities. In higher education, this theory offers a valuable framework for identifying and understanding the factors that contribute to effective learning outcomes [21]. It highlights the importance of various elements, such as teaching methods, student motivation, collaboration, and institutional support, all of which function together within authentic learning contexts [22]. CLT is retained as the primary theoretical lens because it conceptualises learning success as an emergent property of interactions between learners, instructors, and learning environments, which directly matches this study’s aim to examine how multiple pedagogical and motivational conditions combine to produce high learning outcomes. This research aims to identify and analyse the specific configurations of factors that contribute to successful learning outcomes for students in higher education. This goal closely aligns with Constructivist Learning Theory, which emphasises that learning is a dynamic, multifaceted process influenced by active learner engagement within their social and instructional contexts. By framing this study within Constructivist Learning Theory, it transcends simple linear cause-and-effect models and examines how various conditions interact to influence learning outcomes. This theoretical foundation supports the application of a configurational approach that captures the interactive, learner-centred aspects of educational success as envisioned by constructivism. From this perspective, instructional conditions are not expected to operate independently; rather, they function as jointly enabling resources, and different constellations of such resources may yield comparable learning outcomes. This assumption provides a coherent theoretical justification for using a configurational approach to model causal complexity in higher education learning processes [17,14].

To strengthen the scientific basis of the model, the causal conditions included in this study are conceptualised as constructivist learning resources. Teacher-related conditions represent instructional and organisational resources that shape the quality of scaffolding, social interaction, and authentic task design, while student motivation represents the learner’s internal resource that energises engagement and self-regulation. The present framework, therefore, captures both instructional capacity and learner agency, two core pillars of constructivist explanations of learning effectiveness [23].

### 2.2 Outcome variable: Learning outcomes (LO)

Learning outcomes are defined statements that outline what learners should be able to consider, comprehend, and achieve following specific learning experiences [24,1]. Various terms have been used globally to describe learning outcomes, including learning objectives, competencies, learning goals, intended learning outcomes, and assertions [25]. Nevertheless, this discrepancy inherently reflects diverse educational environments, philosophies, strategies, or cultures, making this conceptual mediation relevant, as it addresses the intention behind the ‘learning outcomes’ semanteme [26,27,28]. LO is selected as the focal outcome because it is the most direct indicator of educational effectiveness and is central to curriculum alignment, pedagogy, and assessment decisions in higher education, making it theoretically meaningful and practically actionable.

Theoretically, learning outcomes should indicate observable, significant, and manageable learning gains that align with academic taxonomies, such as Bloom’s hierarchy [29,30]. In this context, learning involves the acquisition, reinforcement, transformation, or extension of behavioural, practical, cognitive, or affective states [31]. However, rooted in mental and constructivist perspectives, learning is inherently an internal, individual, and often implicit process, making it unpredictable and, to some extent, unmanageable [32,33]. In contrast, outcomes are seen as tangible, observable manifestations of learning, often defined for assessment and accountability purposes [24]. Nonetheless, the debate continues regarding whether teaching itself can be equated entirely with performance. Some scholars propose a dual conceptualisation: in the lower-order sense, learning refers to measurable outputs, such as knowledge or skills acquired, while in the higher-order sense, it encompasses metacognitive dimensions, such as intention, self-regulation, and perception of learning processes under the learner’s control [34]. This dual conceptualisation is consistent with a configurational view: high LO may emerge through combinations that emphasise structured instructional design and feedback, or through combinations that strengthen autonomy, motivation, and self-regulation, implying multiple routes to comparable performance.

In recent years, the concept of learning outcomes has emerged as a central theme in discussions about educational innovation and assessment reform [35,36]. This concept gained prominence through the broader outcomes-based education (OBE) movement, which initially emphasised articulating specific learning results as the foundation for designing curricula, pedagogy, and assessment strategies [37,38]. Originally, OBE referred to an instructional model driven by specified competencies to be demonstrated upon course completion. However, the concept has evolved into a more holistic, integrated reform framework that extends beyond education to include societal transformation and workforce readiness [39]. In this broader sense, the emphasis on rigid outcome formulations has decreased, and a growing critique suggests that an excessive focus on output metrics may distort pedagogical priorities or reduce educational complexity to measurable artefacts [40]. Despite these concerns, the traditional conception of learning outcomes, clearly defined, observable, and assessable student competencies, continues to provide substantial value for curriculum alignment and assessment innovation. Accordingly, the present study treats LO as an outcome to be explained while explicitly recognising that LO is produced by interacting pedagogical and motivational mechanisms rather than a single dominant factor, which supports both the theoretical framing and the choice of fsQCA as an analytic strategy [17].

### 2.3 Causal conditions

#### 2.3.1 Pedagogical innovation (PI).

Pedagogical innovation has evolved from scholarly interest in instructional practices, particularly between the late 1970s and the early 2000s, a period marked by the emergence of terms such as “teacher behaviour,” “didactic strategies,” and “instructional methods” to articulate new pedagogical perspectives [41,42]. Over time, the definition of pedagogical innovation has expanded to encompass the integration of technological and pedagogical knowledge, especially within the Technological Pedagogical Content Knowledge (TPACK) framework [43,44]. This broadened understanding has been widely explored in educational research, generating sustained debate over the conceptual scope, theoretical underpinnings, and classroom applications of innovation in teaching [45,46,47]. PI is included because constructivist learning requires instructional designs that promote active engagement, problem solving, collaboration, and reflection; pedagogical innovation is one of the main levers through which instructors can redesign tasks and learning experiences to create these conditions.

Throughout the evolution of education, new ideas and systems have consistently acted as catalysts for transformative change. Paradigm shifts occur as evolving conceptions of how learning occurs challenge and ultimately displace traditional educational models [31,48]. These shifts have led to significant improvements in learning environments, instructional design, and pedagogical implementation. Over time, terms such as reform, development, adaptation, transformation, and re-conception have often been used interchangeably to describe what is now commonly referred to as pedagogical innovation in the 21st century [49]. These innovations, which continue to evolve and are subject to scholarly debate, reflect educators’ responses to changing understandings of knowledge, instructional strategies, and learning processes. As technological and disciplinary advancements reshape educational landscapes, concepts once taken for granted are being reexamined. Digital tools, as part of this wave of pedagogical innovation, increasingly redefine teaching practices and are widely believed to enhance literacy and learning outcomes through technology-enhanced instruction [50,51]. There has been a continuous debate regarding the quality of education. In this way, teaching methods are not only essential but also closely linked to learning outcomes [52]. The most commonly used teaching methods include lectures or expositions, questioning, discussion, discovery, working examples, and simulations or modelling [53]. Seminars, tutorials, and laboratory-based activities are also used in conjunction with these methods [54]. Nevertheless, these generic methods can be implemented in various ways, allowing the instructor to utilise individual creativity in tasks [55]. A good teacher is vital in the learning process, as the length of the faculty’s teaching may involve learning [56]. From a resource-based constructivist interpretation, PI is expected to become especially effective when accompanied by teacher efficacy and motivation, because innovation requires confidence to enact new practices and sustained energy to refine them in response to students’ needs.

#### 2.3.2 Teacher leadership (TL).

Teachers’ leadership can be defined in several ways. For instance, it is defined as teachers’ influence on their thoughts, feelings, or actions in ways intended to enhance instructional practice within a school context, thereby strengthening student learning [57,58]. An advantage of this definition is that it emphasises one of the key roles of teachers, the collaborative role, where teachers’ contributions intersect with those of traditional school leaders [59]. It has been asserted that, through this collaborative process, teachers can better identify their professional learning needs and work together to develop a shared language about teaching and learning that is aligned with desired outcomes [60,61]. TL is selected because constructivist learning environments rely on coordinated practices and shared professional norms that sustain active learning and consistent scaffolding across courses and instructors; teacher leadership is therefore an enabling condition that can strengthen the implementation and diffusion of effective pedagogy [62].

Bringing teachers to the centre of leadership aligns with the ongoing changes in school leadership. As traditional hierarchical structures are questioned, new practices focusing on collaboration are being developed to replace them [62,63]. In rigorous evaluations of school leadership practices, it is recommended that school leaders share power to encourage greater teacher involvement [64]. The underlying logic is that teachers’ involvement is essential to achieving successful and sustainable reforms. Thus, a greater emphasis on teacher agency is needed to direct their own professional needs or those of their colleagues [65]. Within a configurational logic, TL may not operate as a stand-alone predictor; its relevance lies in how it combines with innovation and ICT use to create collective capacity, peer support, and coherent instructional change that can support higher LO [66].

#### 2.3.3 Teacher efficacy (TE).

Teacher efficacy encompasses educators’ beliefs in their ability to objectively observe and adjust their teaching practices to enhance student learning, thereby positively influencing students’ educational success. This concept has proven especially relevant to teachers’ pedagogical innovations [67]. In this context, the two categories of teacher efficacy are personal teaching efficacy and general teaching efficacy. The former concerns educators’ beliefs about their ability to foster student learning, while the latter concerns their beliefs about the overall impact of teaching on student learning [68,69,70]. The more efficacious teachers feel, the more likely they are to adopt innovations or discover new applications for existing ideas or materials [70]. TE is included because constructivist teaching requires adaptive scaffolding, responsive feedback, and classroom organisation that enable students to engage in meaningful activities; efficacy beliefs are therefore central to the teacher’s capacity to persist in enacting learner-centred strategies.

Teacher efficacy is a crucial trait with empirical links to effective teaching practices, self-reported student learning, and successful classroom management [71; 72]. Teacher efficacy in teaching with digital technologies positively correlates with teachers’ use of technology in their pedagogical approaches and the educational application of technology within schools [73,74,75]. Teachers who have a higher sense of classroom management efficacy tend to use technology more extensively for educational purposes in their planning, preparation, assessment, and classroom management practices [76]. In this study, TE is theoretically positioned as a foundational resource that can activate other conditions, such as PI and TICT, because high-efficacy teachers are more likely to integrate innovation and technology in ways that remain pedagogically coherent and oriented toward learning outcomes.

#### 2.3.4 Teacher motivation (TM).

Motivation is a crucial concept that has garnered significant attention across all educational levels. Several definitions exist, all highlighting the driving force that propels individuals toward achieving their goals [Ryan & Deci, 2000]. Motivation is typically regarded as dynamic, manifesting through behaviours, effort, and persistence [77]. It exhibits inherent variability, fluctuating throughout the day and influenced by environmental contextual factors [78]. Consequently, it can be assessed at various timescales: state motivation refers to moment-to-moment fluctuations in response to specific stimuli, while trait motivation pertains to enduring aspects [79]. Due to this trait-like quality, the timeframe can be extended significantly, encompassing bonding motivations from early childhood, such as caring for others or contributing to the collective good, and extending to the study of motivational patterns across the lifespan [80]. TM is selected because learner-centred, constructivist instruction is effortful and emotionally demanding; teacher motivation sustains experimentation, persistence, and commitment to active learning practices that can enhance LO.

Teacher motivation is a significant concern for educational leaders and managers because it profoundly impacts student motivation [81]. Driven and engaged teachers often play a pivotal role in advocating for educational improvements and policy advancements, becoming active supporters of students, institutions, and educational authorities [82]. These educators are essential for ensuring that reforms introduced at the policy level are effectively implemented in practice [83]. Moreover, motivation significantly contributes to teachers’ professional satisfaction and sense of purpose. Educators who feel content in their roles generally experience greater happiness and are more committed to their institutions [84]. Such job satisfaction also correlates with reduced absenteeism and lower staff turnover rates, benefiting the overall stability of educational environments [85]. Nonetheless, despite the recognised value of teacher motivation, there remains a stark contrast between its acknowledged importance and its actual prevalence. Motivation remains a foundational element in ensuring successful teaching and learning outcomes. In configurational terms, TM is expected to strengthen the effectiveness of PI and TE by sustaining teachers’ engagement with innovation and by supporting the continuous adjustments required for scaffolding and feedback, which are central to constructivist learning processes.

#### 2.3.5 Teachers’ ICT use (TICT).

The integration of Information and Communication Technologies (ICT) in education is a global imperative for processing and managing the exponential growth of knowledge, thereby enhancing the quality of education and pedagogical outcomes [86]. The shift toward a knowledge-based society and the use of digital technology for exchanging the world’s knowledge are accelerating [87]. Communication technology is a vital means of access to an information-based society. Recent studies show that, when used appropriately, ICT can help complement and strengthen the relevance of education in a rapidly expanding, knowledge-based, and networked society, thereby raising the quality of education by making learning and teaching an active, complex, and coherent process connected to real life [66]. Pedagogically, the adoption and use of ICT in schools lead to significant educational and pedagogical outcomes, which include expanding pedagogical resources and facilitating knowledge sharing and networking, making lessons more engaging, helping teachers simplify complex situations, clarifying concepts and principles, guiding attention and sources, aiding learners in reinforcing material, providing multi-modal presentations that cater to the diverse learning styles of individual learners, supporting learners with particular needs, promoting collaborative learning, tracking homework of students, ensuring that learners without stable home environments are accommodated, and creating a digital world with new ways of knowledge sharing as an outcome of collaboration or learning [88]. Despite acknowledging these benefits, studies indicate that many schools utilise technologies that are often underutilised, and few institutions fully leverage the teaching and learning potential of ICT [89]. TICT is included because, from a constructivist perspective, ICT can provide rich learning resources, collaboration spaces, and multimodal scaffolds that support active learning, but its effects are expected to depend on pedagogical alignment rather than mere access or frequency of use.

#### 2.3.6 Student motivation (SM).

**Fig 1:**
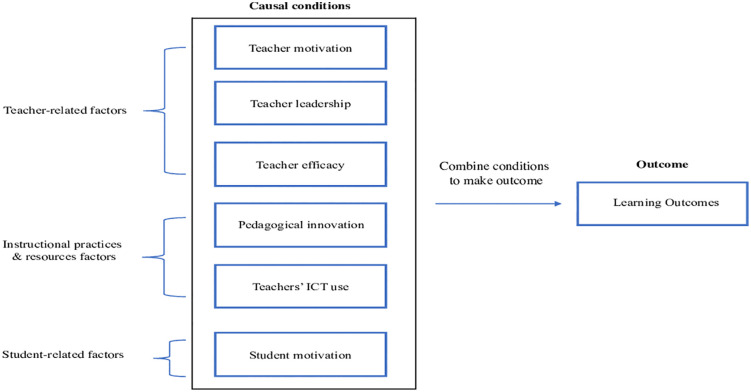
Research model.

Motivation is a key driver of student performance, achievement, and learning [90]. Research shows that student motivation often declines significantly in higher education [91]. Therefore, as we enhance instruction, we must focus not only on content and pedagogy but also on the timely and critical component of student motivation. Instructional innovations that foster student motivation have been overlooked and deserve increased attention [75]. In the highest-quality learning environment, knowledge is readily accessible; learning, when pursued, is both rewarding and fulfilling, and the emotions that drive curiosity and interest spark the desire to explore various information avenues [92]. The rapid pace of societal advancement, driven by an unprecedented increase in human brain capacity, requires learners to be aware of the processes, practices, and technologies necessary to keep pace with this progress [93]. One of the most active areas of research in educational psychology, which influences pedagogical innovation aimed at meeting the educational needs of high achievement, inquiry, and challenge in the 21st century, is student motivation, any internal or external process that energises or directs goal-oriented activities [94]. Motivation is a primary predictor of performance and achievement, particularly academic achievement [95]. It serves as a key driver of learning, acquisition, determination, attention, participation, and effort, especially in high-mental-demand academic tasks [96,97]. Therefore, it is essential that as instructional methods and materials are developed, enhanced, or implemented to address the content to be taught, the planned structure, characteristics, and delivery of instructional activities also provide insight into how these methods or approaches maximise student motivation, engagement, and learning efficiency [23]. SM is selected because constructivist learning requires active participation and self-regulation; student motivation is the internal mechanism that sustains effort, attention, and persistence, thereby enabling students to benefit from instructional resources such as innovation, leadership, efficacy, and ICT-supported activities. Based on the literature, Fig 1 illustrates our research model, and the hypothesis is formulated as follows:

**Hypothesis**: Different configurations of causal conditions can lead to high learning outcomes.

## 3. Methodology

### 3.1 Data collection and sample

Data collection is a crucial step in the in-depth analysis of the configurations that lead to student learning outcomes. This is especially true when considering the importance of the methodology employed, particularly through the Qualitative Comparative Analysis (fsQCA) approach. To successfully execute this essential phase and ensure effective information gathering, a detailed questionnaire was designed and distributed to Moroccan students. This questionnaire aims to collect relevant, meaningful, and diverse information to illuminate learning dynamics and enhance understanding of the factors influencing students’ academic performance.

A consent-to-participate form was attached to the questionnaire to obtain written approval, and the study received ethical approval from the Faculty of Economics and Management, Beni Mellal, Morocco (Ref. 08.02.CE25/FEMBMM). All participants were provided with clear information about the study’s purpose and procedures, and gave informed consent prior to participation, signed, and completed the questionnaire. Participation was voluntary, and all data were handled with strict confidentiality and anonymisation measures to protect participants’ privacy, in line with internationally recognised ethical principles for research involving human participants.

A survey design was selected because the study focuses on latent perceptions and self-reported experiences (e.g., motivation, efficacy, leadership, ICT use) that are best captured through standardised psychometric scales. The instrument was compiled from established measures to ensure conceptual fit with the constructs and comparability with prior research, while allowing for set calibration in fsQCA [17]. Prior to full deployment, items were reviewed for clarity and contextual relevance, and a brief pre-test with students was used to refine wording and reduce ambiguity. The sample consists of 349 students enrolled in various types of higher education institutions. These students are taking courses that may be delivered online, in person, or in a hybrid format that combines both. This heterogeneity increases the likelihood of multiple effective pathways to high learning outcomes, consistent with configurational reasoning.

### 3.2 Measurement instrument

In this comprehensive research framework, the carefully selected items used to measure each variable have all been validated in the existing literature. Table 1 provides detailed information for each specific condition, listing the precise items drawn from prior studies along with their relevant references. All items were measured on a 7-point Likert scale. Tools were selected using three criteria: theoretical alignment with construct definitions, prior evidence of reliability and validity, and suitability for adaptation to the Moroccan higher education context without changing item meaning. After adaptation, psychometric quality was assessed through internal consistency (Cronbach’s alpha and composite reliability), convergent validity (item loadings and AVE), and discriminant validity (Fornell–Larcker and/or HTMT). Items showing weak performance were examined and retained only when theoretically justified; otherwise, they were refined or removed to strengthen measurement precision. Validated scores were then calibrated into fuzzy-set membership using transparent anchors (full membership, crossover, full non-membership), ensuring that the measurement step supports robust and generalizable fsQCA results [17].

**Table 1:** Measurement instruments.

Constructs	Number of Items	Sources
Learning outcomes	2	Schmitz, B., & Wiese, B. S. [98].
Pedagogical innovation	5	Carvalho et al. [99] Haleem et al. [100]
Teacher leader	8	Sugg, Sally Ann, [101],
Teacher efficacy	7	West, C. et al. [102]
Teacher motivation	5	West, C. et al. [102]
Teachers’ ICT use	3	O’Dwyer et al. [103]
Student motivation	3	Laura F.N. [104]

## 4. Results

### 4.1 Descriptive statistics

The descriptive statistics show that PI is very high (M = 5.26; SD = 0.51), indicating that teachers view the new pedagogical approaches very positively. TM also scores high (M = 5.16; SD = 0.43), reflecting continued engagement in their teaching practices. TE has a more moderate mean (M = 4.16; SD = 0.75), highlighting greater variability in perceptions of their classroom performance. LO is strong (M = 4.74; SD = 0.83), indicating that the adopted methods yield strong learning outcomes for students. TL shows a lower mean (M = 3.43; SD = 1.04), indicating differences in practising the pedagogical leadership role. SM is at an intermediate level (M = 2.80; SD = 0.96), implying variations in student engagement. Finally, the use of TICT is moderate (M = 3.49; SD = 0.82), showing partial adoption of information and communication technologies. The lack of missing data and the stability of the minimums and maximums confirm the quality and consistency of the sample, making it suitable for further analysis (see Table 2).

**Table 2:** Descriptive statistics.

Variable	Moyenne	Écart‑type	Minimum	Maximum	N cas	Missing
PI	5,2550	0,5073	1	6	349	0
TM	5,1597	0,4321	3,75	6	349	0
TE	4,1572	0,7543	1	6	349	0
LO	4,7364	0,8297	1,5	6	349	0
TL	3,4327	1,0423	1,25	5	349	0
SM	2,7966	0,9571	1	5	349	0
TICT	3,4928	0,8202	1	5	349	0

### 4.2 Exploratory Factor Analysis

Exploratory Factor Analysis (EFA) is a statistical method for revealing the underlying structure of a large set of observed variables. It assists researchers in identifying latent constructs or factors that account for the correlation patterns among observed items, all without enforcing a predefined structure.

The EFA using JASP software, reveals a clear seven‐factor structure: Factor 1 strongly loads all TL items (0.755–0.830), Factor 2 the TE items (0.494–0.664), Factor 3 the SM items (0.679–0.961), Factor 4 the TICT items (0.654–0.891), Factor 5 the TM items (0.451–0.672), Factor 6 the PI items (0.425–0.627), and Factor 7 the LO items (0.791–0.814). Uniqueness values are all below 0.75 (except for a few TE items), confirming that each item is well explained by its primary factor (see Table 3).

**Table 3:** Factor loadings.

Item	Factor 1	Factor 2	Factor 3	Factor 4	Factor 5	Factor 6	Factor 7	Uniqueness
TL1	0.830							0.311
TL2	0.821							0.317
TL3	0.820							0.324
TL8	0.810							0.336
TL4	0.767							0.404
TL7	0.763							0.416
TL5	0.756							0.409
TL6	0.755							0.423
TE1		0.664						0.556
TE2		0.622						0.586
TE7		0.614						0.612
TE5		0.552						0.679
TE6		0.549						0.688
TE3		0.531						0.703
TE4		0.494						0.741
SM2			0.961					0.120
SM3			0.868					0.216
SM1			0.679					0.444
TICT1				0.891				0.262
TICT2				0.694				0.439
TICT3				0.654				0.522
TM1					0.672			0.575
TM2					0.607			0.664
TM3					0.570			0.625
TM4					0.501			0.726
TM5					0.451			0.709
PI1						0.627		0.617
PI2						0.614		0.666
PI4						0.571		0.615
PI5						0.497		0.677
PI3						0.425		0.806
LO1							0.814	0.335
LO2							0.791	0.356

The Chi-squared test yields χ² = 619.892 with 318 degrees of freedom (df) and a p-value < 0.001. This p-value, below the typical threshold of 0.05, indicates that the model is statistically significant. The overall KMO value is 0.796, indicating adequate sampling adequacy for factor analysis. Most items have values above 0.6, supporting their inclusion in the study. Bartlett’s test of sphericity reports a chi-square value of χ² = 4678.643 with 528 degrees of freedom and a p-value less than 0.001. This highly significant result suggests the correlation matrix is not an identity matrix. In other words, there are enough correlations among variables to justify factor analysis. The test confirms that the dataset is appropriate for dimensionality reduction.

### 4.3 Correlation plot

**Fig 2:**
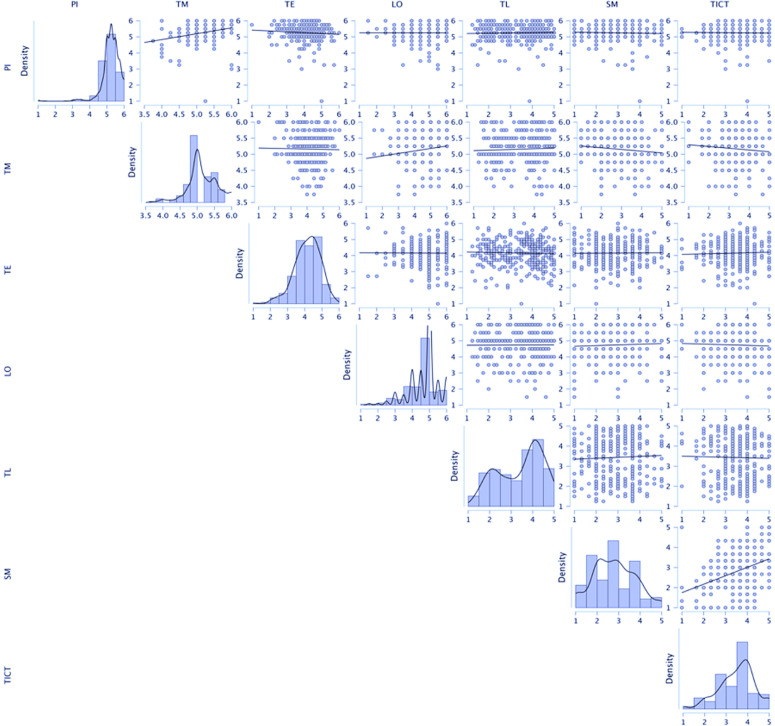
Densities and scatter plots of the variables.

The bivariate scatterplots and diagonal histograms display each dimension’s univariate distribution and the pairwise relationships among PI, TM, TE, LO, TL, SM, and TICT. Along the diagonal, PI, TM, and LO show density peaks at the higher end of the scale, indicating generally favourable assessments. In contrast, TL and SM are more dispersed, reflecting varied opinions. In the lower-right panels, the scatterplots confirm the absence of extreme correlations: points are widely scattered, with no tight clusters, indicating that although some pairs exhibit a slight positive trend (notably PI–TM and TE–LO), no strong linear relationship prevails. This pattern supports the discriminant validity of the dimensions, as each captures a distinct aspect of the phenomenon. Overall, the plot highlights the satisfactory internal coherence of the scales (with several variables concentrated at high values) while underscoring the relative independence of the constructs – a necessary condition for subsequent structural modelling analyses (see Fig 2).

### 4.4 Validity, reliability and discrimination

To assess the validity and reliability of the constructs, we used Cronbach’s alpha, outer loadings, and the average variance extracted (AVE). The results in Table 4 show that all items have outer loadings above 0.70, Cronbach’s alpha values exceed 0.70, and AVE values exceed 0.50. Overall, these findings confirm that the measurement indicators meet the recommended thresholds and that all constructs are valid and reliable, providing a solid basis for subsequent analyses.

**Table 4:** Validity and reliability.

	Outer loadings	Cronbach’s alpha	Composite reliability (rho_a)	Average variance extracted (AVE)
LO1 < - LO	0.974	0.949	0.950	0.751
LO2 < - LO	0.976			
PI1 < - PI	0.787	0.770	0.933	0.636
PI2 < - PI	0.721			
PI3 < - PI	0.725			
PI4 < - PI	0.736			
PI5 < - PI	0.709			
PI6 < - PI	0.839			
SM1 < - SM	0.912	0.869	0.885	0.792
SM2 < - SM	0.881			
SM3 < - SM	0.876			
TE1 < - TE	0.711	0.854	0.871	0.627
TE2 < - TE	0.769			
TE3 < - TE	0.790			
TE4 < - TE	0.773			
TE5 < - TE	0.748			
TE6 < - TE	0.795			
TE7 < - TE	0.784			
TICT1 < - TICT	0.831	0.835	0.881	0.748
TICT2 < - TICT	0.895			
TICT3 < - TICT	0.867			
TL1 < - TL	0.743	0.721	0.964	0.655
TL2 < - TL	0.737			
TL3 < - TL	0.722			
TL4 < - TL	0.753			
TL5 < - TL	0.790			
TL6 < - TL	0.727			
TL7 < - TL	0.744			
TL8 < - TL	0.748			
TM1 < - TM	0.791	0.788	0.841	0.685
TM2 < - TM	0.859			
TM3 < - TM	0.832			
TM4 < - TM	0.778			
TM5 < - TM	0.761			

To assess discriminant validity, we applied the Fornell–Larcker criterion, which involves comparing, for each construct, the square root of the AVE (placed on the diagonal of the table) with the correlations with the other constructs (off-diagonal). Discriminant validity is established when the diagonal value for each construct is strictly greater than all of its correlations with the other constructs, indicating that the construct shares more variance with its own items than with the other variables in the model. The results in Table 5 show that the Fornell–Larcker criterion is satisfied. This indicates that each construct shares more variance with its own indicators than with the other variables, confirming that the constructs are distinct and supporting discriminant validity.

**Table 5:** discriminant validity.

	LO	PI	SM	TE	TICT	TL	TM
LO	0.867						
PI	0.836	0.797					
SM	0.535	0.648	0.890				
TE	0.576	0.559	0.619	0.792			
TICT	0.768	0.637	0.606	0.675	0.865		
TL	0.662	0.547	0.640	0.502	0.576	0.809	
TM	0.518	0.522	0.588	0.666	0.639	0.638	0.828

### 4.5 fsQCA

The fsQCA methodology emerges as a transformative tool for deciphering complex causality in educational research contexts [105,106], particularly in exploring configurations that promote successful student learning outcomes. Originating from the broader framework of Qualitative Comparative Analysis, fsQCA enhances traditional binary analysis by incorporating fuzzy logic, which allows for varying degrees of membership in defined sets. This nuanced approach enables researchers to model configurations as combinations of necessary and sufficient conditions, thereby providing a more sophisticated lens for examining the multifaceted nature of educational phenomena.

The choice of fsQCA is particularly appropriate for the present study, as the research design seeks to capture complex, non-linear, and equifinal relationships between pedagogical, technological, and motivational factors and learning outcomes. Unlike symmetric statistical techniques, fsQCA enables the identification of multiple causal paths leading to the same outcome, aligning closely with the study’s research questions and hypotheses, which focus on configurational effects rather than isolated net impacts.

#### 4.5.1 Calibration.

Data calibration corresponds to the data-coding phase and involves converting variables into continuous values between 0 and 1. This process requires specifying full membership (value = 1), full non-membership (value = 0), and the crossover point (value = 0.5) [17]. The crossover point serves as the qualitative anchor at 0.5, representing the threshold at which it becomes impossible to determine whether the variable is more a member or a non-member of the set. We then define threshold values close to full membership (e.g., 0.95) and full non-membership (e.g., 0.05) [Woodside, 2013]. These calibration thresholds were selected based on both theoretical considerations and the empirical distribution of the data, ensuring consistency between substantive knowledge and observed values. This approach enhances the interpretability and robustness of the calibrated sets, particularly given the ordinal nature of the survey data.

More precisely, the FsQCA software calculates a calibration score for each case using these three anchors (0.05, 0.50, 0.95). As presented in Table 6.

**Table 6:** Data calibration.

	Descriptive Statistics			Calibration Criteria		
	Mean	Min	Max	95%	50%	5%
PI	5,25	1	6	5	3	1
TM	5,15	3,75	6	6	5	4
TE	4,15	1	6	5	3	1
LO	4,73	1,5	6	5	3,5	2
TL	3,43	1,25	5	5	3,5	2
SM	2,79	1	5	5	3	1
TICT	3,49	1	5	5	3	1

#### 4.5.2 Analysis of necessary conditions.

In the initial phase of fsQCA, the analysis aims to determine whether certain causal conditions are essential for the outcome to occur [107]. A condition is considered necessary when its presence (or absence) is systematically required for the presence (or absence) of the outcome [108]. To assess the necessity of each of the six conditions, the consistency score serves as the primary criterion. According to Ragin [109], a condition is deemed necessary if its consistency exceeds 0.90 and it is regarded as almost always necessary if the score falls between 0.80 and 0.90.

The analysis of causal conditions associated with the outcome variable LOc (Learning Outcomes), shown in Table 7, reveals several significant patterns. Pedagogical innovation (PIc) stands out with very high consistency (0.986) and considerable coverage (0.877), emphasising its vital role in configurations that lead to strong learning outcomes. Conversely, its absence (~PIc) shows very low consistency, confirming that it is rarely associated with satisfactory results.

**Table 7:** Necessary conditions analysis.

Causal condition	Outcome Variable: LOc	
	Consistency	Coverage
PIc	0.986077	0.876647
~PIc	0.051610	0.999347
TMc	0.645845	0.928741
~TMc	0.456127	0.948147
TEc	0.860374	0.909943
~TEc	0.225215	0.975186
TLc	0.556177	0.931831
~TLc	0.529715	0.913924
SMc	0.495331	0.951808
~SMc	0.606304	0.924160
TICTc	0.706894	0.929851
~TICTc	0.395247	0.949546

Note: The tilde symbol (~) before the causal condition represents the absence of the condition.

Teachers’ perceived efficacy (TEc) also demonstrates high consistency (0.860), indicating that it is almost always necessary in configurations that result in positive outcomes, whereas its absence is rarely involved. The use of information and communication technologies by teachers (TICTc) also shows a notable relationship (consistency = 0.707), suggesting that effective ICT use enhances learning.

In contrast, conditions such as teacher motivation (TMc), teacher leadership (TLc), and student motivation (SMc) exhibit more moderate consistency levels, suggesting they may play a supporting or contextual role in specific configurations but are not consistently decisive on their own.

Taken together, these results provide empirical support for the study’s first research question by identifying pedagogical innovation and teacher efficacy as critical foundational conditions for achieving high learning outcomes, while also highlighting the contingent role of other factors within broader causal configurations.

#### 4.5.3 Analysis of sufficient conditions.

To analyse sufficient conditions, it is essential to construct, adjust, and interpret a truth table for each outcome of interest [17]. A truth table contains 2^k rows, where k represents the number of causal conditions, with each row reflecting a unique combination of those conditions. Based on set membership scores, each case is assigned to a specific configuration, enabling the fsQCA algorithm to generate a corresponding truth table for the outcome. In this study, a truth table was produced for the LO outcome. To improve the reliability of the analysis, two thresholds were applied: a consistency cutoff and a Proportional Reduction in Inconsistency (PRI) threshold [17]. The minimum consistency score was established at 0.80, while the PRI threshold was set at 0.70. Incorporating the PRI criterion helps maintain the solutions’ distinctiveness by ensuring that a given configuration does not simultaneously lead to both the outcome and its negation.

These threshold choices are consistent with established methodological recommendations and ensure that the identified configurations exhibit both empirical relevance and theoretical plausibility.

Table 8 combines both parsimonious and intermediate solutions using the symbolic notation system introduced by Fiss [14]. Each column represents a distinct configuration. A filled black circle (●) signifies the presence of a condition, while a crossed-out circle (⊗) indicates its absence. Blank cells imply that the condition is not decisive in that configuration. Larger circles correspond to core conditions—those familiar to both types of solutions—while smaller circles reflect peripheral conditions, appearing only in the intermediate solution. To identify which solution corresponds to each outcome, configurations are labelled accordingly (e.g., “LOc 1”). It is noteworthy that PIc (perceived pedagogical innovation) plays a consistently central role across all configurations. Meanwhile, other factors, such as TICTc (teachers’ ICT use) and TMc (teacher motivation), vary by path. The raw and unique coverage, along with the consistency values, confirm the reliability of these configurations in explaining the observed outcome, with a high overall solution coverage (0.875) and strong consistency (0.911).

**Table 8:** Configurations for a high degree of learning outcomes.

	Learning outcomes				
	LOc 1	LOc 2	LOc 3	LOc 4	LOc 5
TLc		⊗	•	•	•
SMc		⊗	⊗		•
TICTc			•	•	•
TEc	●				●
TMc		•		•	•
PIc	●	●	●	●	
Raw Coverage	0.853227	0.325805	0.322670	0.361605	0.275139
Unique Coverage	0.336625	0.007787	0.001247	0.006135	0.002124
Consistency	0.910959	0.957500	0.943426	0.946946	0.973870
Solution Coverage	0.875476				
Solution Consistency	0.911007				

The existence of five distinct configurational paths leading to high learning outcomes clearly illustrates the principle of equifinality and provides strong empirical validation for the study’s configurational hypotheses. Therefore, the research hypothesis is confirmed, demonstrating that high learning outcomes can be achieved through multiple, equally effective combinations of pedagogical, technological, and motivational conditions.

**Fig 3:**
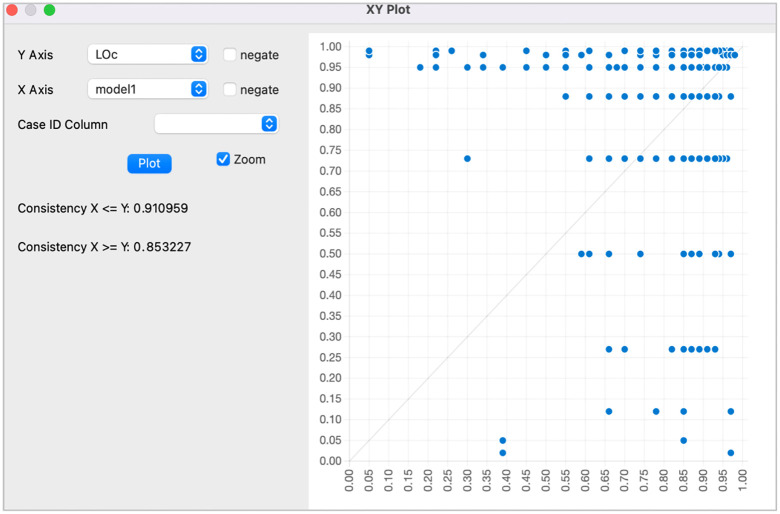
Alignment between Model 1 and LOc1 (XY Plot).

The XY plot 3 (Fig 3) illustrates the relationship between the degree of membership of cases in the selected causal configuration (X-axis: Model 1) and their membership in the outcome under study (Learning Outcomes – LOc, Y-axis). A significant clustering of cases appears in the upper-right quadrant, indicating that cases with a high degree of membership in the causal combination (Model 1) also tend to exhibit a high level of the outcome. This distribution suggests a strong sufficiency relationship between the causal configuration and the outcome, supported by a high consistency score (X ≤ Y = 0.910959) and substantial coverage (X ≥ Y = 0.853227). The near absence of cases in the upper-left or lower-right quadrants indicates that few cases violate this relationship, thereby reinforcing the model’s empirical validity.

By visually confirming the alignment between the configurational results and the outcome, Fig 3 strengthens the interpretability of the fsQCA findings and provides an intuitive link between the statistical results, the research hypotheses, and the underlying theoretical framework.

## 5. Discussion

This study highlights the intricate nature of pedagogical dynamics that significantly impact learning outcomes across various educational environments. It shows that improving students’ academic performance relies not on isolated factors but on a combination of interconnected conditions, including teacher motivation, effective leadership, educators’ and students’ self-efficacy, and high levels of student engagement. These elements interact closely to foster a supportive and nurturing environment that promotes effective learning, underscoring the urgent need for a comprehensive approach to understanding and analysing academic performance. Interpreted critically, this pattern suggests that learning outcomes are best understood as an emergent result of causal complexity, in which conditions reinforce one another, and no single factor can be assumed to be universally decisive across contexts [17,14]. This insight helps clarify why earlier studies sometimes produce inconsistent or weak net effects: when variables are examined independently, their impact may appear unstable, whereas configurational analysis reveals the specific contexts in which they become effective. This new approach differs from many previous studies that have attempted to identify the determinants of learning outcomes’ success using a linear, unidimensional approach [91,88,75]. Accordingly, the study contributes to the literature by moving from “average effects” to “effective pathways,” demonstrating that distinct combinations of teacher and student resources can yield similarly high performance, thereby strengthening the explanatory realism of research on higher education learning outcomes [15].

The integration of pedagogical innovation and information and communication technologies (ICT) into teaching practices serves as a crucial tool for transforming and enhancing educational processes across various settings. This idea aligns with Tondeur et al. [66] and Ukpe [110]. Their impact is significant when supported by motivated, confident teachers who can provide effective, constructive leadership in their classrooms. Additionally, as suggested by Howard et al. [75], the findings confirm that student motivation plays a key role as a catalyst, not only enhancing but also broadening the positive effects of these institutional and pedagogical factors, resulting in a livelier and more engaging learning environment. Contrary to these studies that endorsed an exclusive impact of these variables, our results demonstrate that leveraging both innovative teaching methods and modern technologies is essential to creating a vibrant educational ecosystem that motivates both teachers and students. Importantly, the findings nuance technology-centred expectations by showing that ICT and innovation are not “automatic solutions”; their contribution becomes salient when embedded in a human-capacity configuration characterised by teacher efficacy, sustained motivation, and constructive leadership. This provides a theoretically grounded explanation for divergences in prior evidence, in which technology effects were sometimes positive and sometimes negligible, depending on whether pedagogical integration and teacher readiness were present.

One of the most interesting aspects of this research is the opportunity for an integrative, sustainable approach, suggesting that sustainable improvements in learning outcomes depend on combining multiple conditions rather than isolated actions. Educational policymakers are therefore encouraged to develop integrated strategies that simultaneously foster pedagogical innovation, ongoing teacher training, and active student participation. Such a comprehensive approach is essential to effectively address current educational challenges and enhance overall academic quality. From an applied standpoint, the study translates evidence into implementable priorities: professional development should jointly target pedagogical design and efficacy-building, leadership initiatives should strengthen collaborative teacher leadership to support diffusion of innovation, and student support mechanisms should sustain motivation through autonomy-supportive practices and feedback, because motivation amplifies instructional investments.

## 6. Conclusion

This research contributes to the growing body of scholarship examining how pedagogical, motivational, and technological factors intersect to impact student learning outcomes in higher education. Using Constructivist Learning Theory and the fsQCA method, the findings indicate that high academic achievement stems from specific combinations of factors, including teacher motivation, pedagogical leadership, ICT integration, and student engagement, rather than from isolated variables. These results emphasise the need for a configurational approach to educational improvement, moving beyond linear models to better address the complex, context-dependent nature of teaching and learning. The insights from this study provide a solid foundation for developing comprehensive, evidence-based policies and interventions that address the various challenges in higher education. By understanding how human, pedagogical, and technological factors interact, institutions can more effectively promote equity, innovation, and excellence in academic achievement.

## 7. Practical and theoretical implications

The study’s findings have significant implications for educational policymakers, teachers, and researchers. Practically, they emphasise the importance of professional development programs that enhance teachers’ motivation, effectiveness, and leadership skills. It is also essential to promote the use of pedagogical innovations and digital tools in everyday teaching while fostering an academic environment that motivates students. Theoretically, the study supports an integrated approach that goes beyond analysing isolated factors to achieve a comprehensive understanding of the causal configurations that drive optimal learning outcomes. By intersecting the individual, pedagogical, and technological dimensions, this research enhances existing frameworks for analysing educational performance. The key theoretical contribution is to empirically operationalise constructivist assumptions using a configurational method, demonstrating equifinality and conjunction in higher education learning processes and thereby addressing the gap left by predominantly linear research designs. Overall, the study advances knowledge by explaining not only what matters, but what works together, strengthening both the scientific value and the applied relevance of research on learning outcomes.

## 8. Limitations and directions for future research

The current study has several notable limitations that should be recognised, as they hinder the generalizability of the results. First, the study’s exclusive focus on institutions in Morocco raises questions about whether similar outcomes would occur in other contexts, such as other nations or educational fields. Future research could examine the consistency of these findings across a broader range of settings using appropriate data collection methods and more varied comparative samples, thereby enhancing our understanding of the results. Second, while student perspectives and evaluations are crucial indicators of instructional effectiveness, other factors related to student behaviours, activities, and engagement may also contribute to disparities in learning outcomes. It is recommended to use other appropriate statistical or predictive techniques to expand the existing analysis of the effectiveness of higher education programs. This investigation could yield further insights and enrich our comprehension of the involved dynamics.

## Supporting information

S1 Database Location of the data: Data is available at: https://doi.org/10.6084/m9.figshare.31079044. (XLSX)
